# Supplementary material for: Toxic Effects and Mechanisms of Silver and Zinc Oxide Nanoparticles on Zebrafish Embryos in Aquatic Ecosystems
Source: Nanomaterials (Basel). 2022 Feb 21;12(4):717. doi: 10.3390/nano12040717 (PMC8880218; doi:10.3390/nano12040717)
Supplement: Supplementary file 1 [file nanomaterials-12-00717-s001.zip › nanomaterials-1595874-supplementary.pdf]

Supplementary Materials

Toxic Effects and Mechanisms of Silver and Zinc Oxide Nanoparticles on Zebrafish Embryos in Aquatic Ecosystems

Yen-Ling Lee [1,2,†], Yung-Sheng Shih [1], Zi-Yu Chen [1,†], Fong-Yu Cheng [3], Jing-Yu Lu [1], Yuan-Hua Wu [4,*] and Ying-Jan Wang [1,5,*]

1 Department of Environmental and Occupational Health, College of Medicine, National Cheng
Kung University, Tainan 70428, Taiwan; yenpig8291@gmail.com (Y.-L.L.); a08916@yahoo.com.tw (Y.-S.S.); q781001@gmail.com (Z.-Y.C.); annie880308@gmail.com (J.-Y.L.)

2 Department of Oncology, Tainan Hospital, Ministry of Health and Welfare, Tainan 70101, Taiwan

3 Department of Chemistry, Chinese Culture University, Taipei 11114, Taiwan; zfy3@ulive.pccu.edu.tw

4 Department of Oncology, National Cheng Kung University Hospital, College of Medicine, National Cheng Kung University, Tainan 70428, Taiwan

5 Department of Medical Research, China Medical University Hospital, China Medical University,
Taichung 40402, Taiwan

* Correspondence: wuyh@mail.ncku.edu.tw (Y.-H.W.); yjwang@mail.ncku.edu.tw (Y.-J.W.);
Tel.: +886-6-235-3535 (ext. 5804) (Y.-J.W.)

† These authors contributed equally to this work.

Spiking AgNPs or ZnONPs into natural water samples led to significant acute toxicity to zebrafish embryos, whereas the level of acute toxicity was relatively low when compared to Milli-Q (MQ) water, indicating the interaction and transformation of AgNPs or ZnONPs with complex components in a water environment that led to reduced toxicity.

Compared with the positive control group (3,4-DCA), the original water samples that were not spiked with AgNPs and ZnONPs did not cause obvious mortality at 24, 48, and 96 hpf. Even in the sample from the polluted Erren River, the survival rate was still high (Figure S1a–d). When the original water samples were filtered through membranes with different pore sizes of 1 μm, 0.45 μm, 0.22 μm, and 0.1 μm, the filtrates also did not produce significant adverse effects. The survival rates of all groups were still greater than 80% (Figure S1a–d).

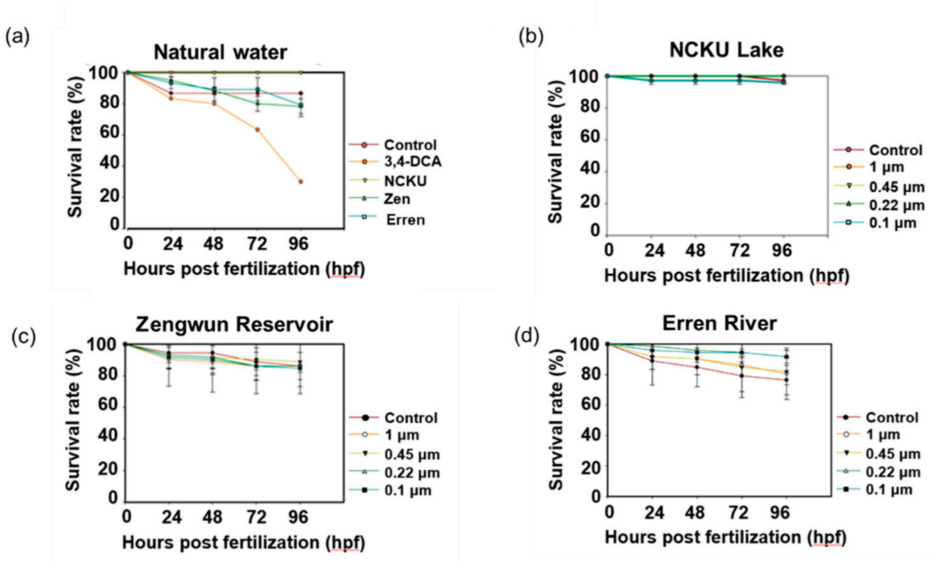

Figure S1. Survival rate of zebrafish embryos exposed to original water. (**a**) The survival rate of embryos treated with original water of NCKU Lake, Zengwun Reservoir, and Erren River. Survival rates of each natural water group were high. Four mg/L 3,4-dichloroaniline (3,4 DCA) was the positive control of acute embryo toxicity assay. Survival rate of zebrafish embryos exposed to original water after passing through 1 μm, 0.45 μm, 0.22 μm, and 0.1 μm filters of (**b**) NCKU Lake, (**c**) Zengwun Reservoir, and (**d**) Erren River at 0, 24, 48, 72, and 96 hpf. hpf, hours post-fertilization.

The filtrates of water samples from NCKU Lake, Zengwun Reservoir, and Erren River that were passed through 1 μm, 0.45 μm, 0.22 μm, and 0.1 μm filters did not alter the body length of the zebrafish compared with the control group (MQ water). The body length of the zebrafish even increased after exposure to filtrates of natural water compared to the control group, but significant differences were not observed between the filtrate groups.

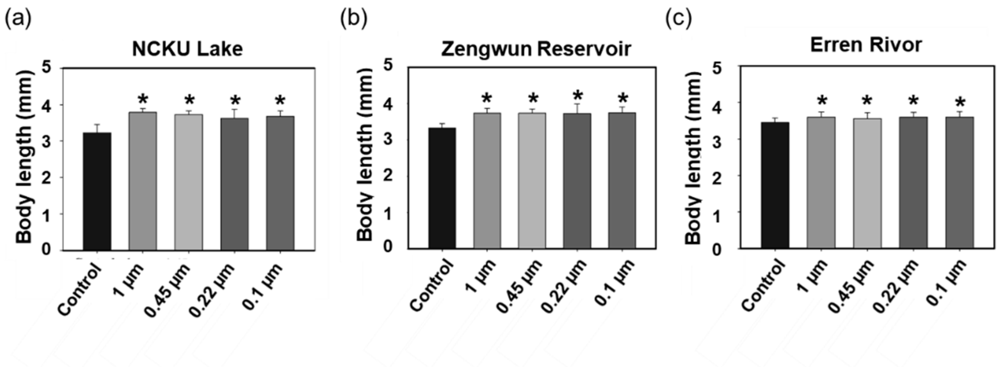

Figure S2. Body length of zebrafish embryos in original water. Body length of zebrafish embryos exposed to original water after passing through 1 μm, 0.45 μm, 0.22 μm, and 0.1 μm filters with different natural water: (**a**) NCKU Lake, (**b**) Zengwun Reservoir, and (**c**) Erren River at 72 hpf. Body length was significantly longer than MQ water in each natural water (* $p \leq 0.05$). Body length of zebrafish embryos were measured by View 7 software. hpf, hours post-fertilization.
